# Displacement, Violence, and Mental Health: Evidence from Rohingya Adolescents in Cox’s Bazar, Bangladesh

**DOI:** 10.3390/ijerph18105318

**Published:** 2021-05-17

**Authors:** Katherine O’Connor, Jennifer Seager

**Affiliations:** Department of Global Health, George Washington University, 950 New Hampshire Ave, Washington, DC 20052, USA; ktoconnor96@email.gwu.edu

**Keywords:** Rohingya, refugee, Bangladesh, displacement, trauma, mental health

## Abstract

The Rohingya have endured generations of trauma through displacement and targeted violence in Myanmar. Hundreds of thousands have been forced out of the country, with a large proportion settling in refugee camps in Cox’s Bazar, Bangladesh. This study examines the impacts of exposure to trauma on mental health outcomes among Rohingya adolescents living in Bangladesh. Post-traumatic stress disorder (PTSD) and depression are examined as outcomes. The main explanatory variable is a measure of exposure to trauma at two levels of proximity (experiencing and witnessing). Resilience is investigated as a potential effect modifier. Experiencing and witnessing traumatic events are positively and significantly associated with PTSD and depression. However, this effect is only seen for PTSD as a continuous measure, reflecting high rates of low-level PTSD in this population. Resilience is found to reduce the effects of trauma on depression, indicating an effect modification of this relationship.

## 1. Introduction

According to the United Nations High Commissioner for Refugees (UNHCR), in 2018, there were 25.9 million refugees across the globe, over half of whom are under the age of 18 [1]. Refugee populations across the globe experience exposure to violence and trauma. Studies have shown that refugees who have been victims of human rights violations experience a large number of traumatic events, with individuals reporting between 7 and 15 traumatic incidences [2]. This exposure to trauma has been found to translate into significant mental health issues in a diverse number of populations [3]. Adolescent refugees are particularly at risk due to adolescence being a time of heightened psychosocial vulnerability, being the age of onset of most mental disorders that are likely to persist into adult life [4,5], and the World Health Organization estimates that half of all mental health conditions develop by the age of 14 [6]. Additionally, adolescents deal with other challenges, such as the development of self-identity and independence [7]. The addition of exposure to traumatic experiences during this vulnerable time can be extremely detrimental to future development [8]. Poor mental health is associated with lower educational achievement [9,10,11,12,13,14], lower self-esteem and self-efficacy which influence schooling decisions and later-life wages [15,16], lower quality of life [17], and with increased risky decision making [18,19].

This study uses baseline data from the Gender and Adolescence: Global Evidence (GAGE) and Cox’s Bazar Panel Survey (CBPS) to examine the burden of poor mental health and exposure to trauma among Rohingya adolescents aged 15–18, living in refugee camps in Cox’s Bazar, Bangladesh. In order to provide context for rates of mental health disorders and exposure to trauma among Rohingya adolescents, we provide a descriptive comparison with Bangladeshi adolescents living in Cox’s Bazar nearby the camps. In related research it is found that, pre-displacement, the Rohingya households are comparable to the Bangladeshi households in terms of assets and labor productivity, indicating that the types of stressors that the Bangladeshi adolescents are facing would be similar to those that the Rohingya adolescents would have faced in the absence of persecution in Myanmar and displacement [20]. In addition, we explore the relationship between trauma and mental health outcomes and the role of resilience as a mediator of the relationship between exposure to trauma and mental health outcomes. We find that Rohingya adolescents are exposed to extremely traumatic events at high rates: nearly 50% experience being close to death and over 40% experience being in a combat situation or torture. This exposure to trauma is associated with a 5% increase in PTSD scores and a 30% increase in the likelihood of experiencing major depression. We further find that adolescents with higher resilience are less likely to develop major depression, and may, in fact, reduce the association between exposure to trauma and major depression to zero.

Our contribution to the literature is three-fold. First, we contribute to the limited literature on the burden of mental health disorders among adolescent refugees, and particularly among Rohingya adolescents living in Bangladesh, who have been exposed to extreme violence, death of family members, war, injury and displacement [21]. In other contexts, PTSD, depression, and anxiety have been found to be the most common mental health outcomes in adolescent refugee populations [8]. A study of children and adolescent refugees living in the United Kingdom found that over 25% of the population had experienced significant psychological problems [22]. Additionally, children and adolescents who have experienced war or other traumatic situations have a high chance of experiencing sleep disturbance [23]. In a study on Cambodian refugees resettled in the United States, post-traumatic stress disorder (PTSD) and major depression were the most common mental health outcomes detected. According to this study, 61% of the study population was identified as having PTSD, while 51% were identified as suffering from major depression [2]. An umbrella review of prevalence studies of mental health outcomes in refugee populations also found comparatively high rates of anxiety with PTSD and depression [24].

Second, our research contributes to understanding of the relationship between exposure to violence and mental health outcomes, highlighting nuances that diagnosis of clinical PTSD and depression may miss [25]. Further, post-migratory factors can have a strong effect on mental health outcomes. Post-migration stress, poor socioeconomic conditions, and low levels of social support have all been found to be associated with increased mental health problems in refugee populations [26,27].

Finally, we contribute to the literature on the role of resilience among refugee populations. The literature has found that resilience, defined as personality traits developed following exposure to traumatic events that enable a person to positively adapt to challenging situations, may play a protective role against negative mental health outcomes [28]. Vulnerability and adaptation to risk have been found to be crucial to the development of resilience in the face of traumatic events during childhood. Additionally, exposure to the complexities of the post-migration environment, such as reconfigured family dynamics and school discrimination can affect the development of resilience in refugee children [29,30,31].

The rest of the paper is organized as follows. Section 2 describes the sample and methods, Section 3 presents the results, Section 4 discusses the findings, and Section 5 concludes.

## 2. Methods

### 2.1. Background

The Rohingya people, originally from Myanmar, are a Muslim group persecuted and rendered stateless due to their religion and ethnicity at the hands of the government of Myanmar [32]. While this has been a persistent problem since 1948, with over 250,000 Rohingya fleeing to Bangladesh in the 1990s, a recent arrival of what is estimated to be 745,400 more refugees occurred in 2017 [33,34]. As a result, nearly 1 million Rohingya refugees, over half of whom are under the age of 18, are currently living in 34 refugee camps in the Ukhia and Teknaf Upazilas in Cox’s Bazar, Bangladesh.

### 2.2. Sample

The data for this project were collected by the Gender and Adolescence: Global Evidence (GAGE) longitudinal study nested within the Cox’s Bazar Panel Survey (CBPS). CBPS is a joint undertaking by the GAGE Programme, the Yale Macmillan Center’s Programme on Refugees, Forced Displacement and Humanitarian Responses (PRFDHR), and Poverty and Equity Global Practice of the World Bank. The goal of this partnership is to create a comprehensive dataset on economic, physical, mental health and social outcomes for both refugee and non-refugee populations (see Guglielgmi et al. (2020) for additional information on the sample [35]). This project uses data collected from the first round of the CBPS, which was conducted between March and October of 2019. Within the CBPS, GAGE collected data on 1071 Rohingya adolescents living in 32 camps in Teknaf and Ukhia Upazilas and 1209 Bangladeshi adolescents living within 60 km of refugee camps in 66 communities across seven Upazilas (Teknaf, Ukhia, Chakaria, Ramu, Cox’s Bazaar Sadar, Pekua, and Naikhongchhari). The overall GAGE sample includes adolescents from two age cohorts: younger adolescents (10–14 years in 2019) and older adolescents (15–18 years in 2019). This study focuses on a subsample of 361 Rohingya adolescents aged 15–18 living in camps. In addition, we draw on data from 455 Bangladeshi adolescents aged 15–18 living in host communities near the camps to serve as comparators to the Rohingya adolescents.

### 2.3. Data Collection

For all adolescents, household-level surveys, surveys with selected adult household members, including primary caregivers, and surveys with selected adolescents were conducted. Each adolescent completed a two-part survey that collected information on a range of outcomes to capture adolescent experience according to GAGE’s 3 Cs: Capabilities, Change strategies and Contexts conceptual framework [19] and household and individual level demographic characteristics. In this study, we focus on measures of psychosocial and mental health experiences and capabilities, including exposure to trauma and depression and PTSD symptoms.

All respondents aged 18 years or older and all married adolescents gave verbal consent, and verbal assent was given by all unmarried adolescents under the age of 18, with verbal consent provided by primary caregivers. Surveys were translated into Bangla and delivered in Chittagonian. While the refugee population speaks Rohingya language, Chittagonian as a spoken language is mutually intelligible with the Rohingya language. Each survey enumerator was trained extensively on the administration of each survey and how to work with adolescents. As the survey included sensitive questions, such as on sexual reproductive health and gender-based violence, an effort was made to match the sex of the enumerator to the sex of the adolescent, with an emphasis placed on ensuring female adolescents were surveyed by female enumerators. Ethics approval for this research was obtained from the George Washington University IRB (protocol #071721, approved modification on 27 August 2018) and, in Bangladesh, through Innovations for Poverty Action IRB (protocol #14742 approved on 28 June 2018) in 2018.

### 2.4. Measures

We present findings from a set of indicators that measure adolescent mental health outcomes. Specifically, we use part IV of the Harvard Trauma Questionnaire (HTQ) [36] as a measure of PTSD and the Patient Health Questionnaire-9 (PHQ-9) as a measure of depression. We measure PTSD and the PHQ-9 both as continuous and dichotomous measures based on clinical cut points in the continuous score. While the continuous measures reveal information about the intensity of the symptoms, the dichotomous measure is an indicator of crossing particular thresholds of interest. Interest in both measures may arise if the continuous measure indicates a large share of adolescents with scores near, but just below the cut point of interest.

Part IV of the HTQ, which consists of 16 questions regarding trauma symptom statements (e.g., recurrent thoughts or memories of the most hurtful or terrifying events) with four levels of answers (e.g., not at all, a little, quite a bit, or extremely), was used to create a dichotomous measure of PTSD. All answers were added up to create a composite measure of trauma symptoms. The total score was then divided by the total number of questions answered, creating a score ranging from 1 to 4. Those with a score greater than or equal to 2.5 are classified as experiencing PTSD. This cut-off point was validated as accurate for the clinical diagnoses of PTSD [36].

The PHQ-9 consists of nine questions that measure depression symptoms (e.g., little interest or pleasure in doing things) with four levels of responses worth between 0 and 3 points—not at all (0 points), sometimes (1 point), more than half of the days (2 points), and nearly every day (3 points). Responses to each of the nine questions were summed, with possible final scores ranging from 0 to 27. Two dichotomous measures of depression were then created: a binary indicator for major depression if the PHQ-9 score is greater than or equal to 10 and a binary indicator of at least mild depression if the PHQ-9 score is greater than or equal to 5. The cut-off of 10 for major depression is based on findings of 88% sensitivity and 88% specificity for major depression in the literature [37,38].

Our primary explanatory variable is exposure to trauma as measured by the HTQ Inventory. Each respondent completed a checklist of 12 traumatic events, indicating if they had experienced, witnessed, heard about, or never heard about each event, selecting the most extreme response that is true. The full list of items is shown in Supplementary Table S1 and includes imprisonment, serious injury, a combat situation, rape or sexual abuse, isolation, close to death, forced separation from family, murder of family or friend, murder of strangers, lost or kidnapped, torture, and unnatural death of family or friend. For each level of proximity, we generate an index by summing together the number of events experienced, witnessed, or heard about, and then standardizing the measure to the mean and standard deviation in the sample.

We measure resilience using the Child and Youth Resilience Measure (CYRM), a validated measure with a Cronbach’s alpha of 0.87 [39,40]. The measure consists of 28 statements (e.g., “I know people whom I consider role models for me”) with three potential levels of answers (1 = not at all, 2 = a little/somewhat, 3 = quite a bit/a lot), and measures seven qualitative aspects of resilience: access to material resources, relationships (with family and peers), identity, power and control, cultural adherence, social justice, and cohesion [39]. An index measure of resilience was generated by summing responses to each item and normalizing it to the sample mean and standard deviation, with higher scores indicating higher community support and personality traits associated with resilience.

### 2.5. Empirical Strategy

We employ linear regression to investigate the relationship between experiencing and witnessing trauma and mental health outcomes among Rohingya adolescents. We use both continuous and dichotomous measures of PTSD and depression as our primary outcomes. In all models, we control for age, gender, enrollment in formal or informal education, socioeconomic status of the household (measured using asset deciles based on principal component analysis (PCA) following [41], geographic location, and sampling considerations. We control for enrollment in education and wealth as potential confounders, as access to school programs and greater household resources may be positively correlated with mental health outcomes and also affect the proximity to situations where trauma is likely to arise [42]. We use sample weights to make the estimates representative of adolescents living in our sample areas. We cluster the standard errors at the camp-block level for data from adolescents in camps at a sub-mauza (a geographic administrative district in Bangladesh) for data from adolescents in host communities to adjust for the sampling design.

## 3. Results

### 3.1. Descriptive Statistics

Table 1 presents summary statistics of demographics and outcomes for our sample of Rohingya adolescents (column 1). For comparison, we also include summary statistics of Bangladeshi adolescents living near the camps (column 2). On average, our adolescent Rohingya sample is 16 years old and 51% female. Only 14.8% of the Rohingya adolescents are enrolled in formal or informal schooling due to low access to education in the camps. In contrast, 62% of the Bangladeshi adolescents in this age group are enrolled in school. Among the Rohingya adolescents, 3.7% exhibit signs of PTSD, 12.5% exhibit PHQ-9 scores that suggest major depression, and over the half (55%) of the sample exhibit signs of at least mild depression, while only 40% of the Bangladeshi adolescents do. Although rates of PTSD and major depression are not statistically different between the Rohingya and Bangladeshi adolescents, rates are higher among Rohingya for both, and Rohingya have statistically higher PTSD (*p* < 0.001) and PHQ-9 (*p* = 0.004) index scores, suggesting higher risk of depression and PTSD among Rohingya adolescents.

Notable in Table 1 is that Rohingya adolescents have experienced nearly 3 traumatic events on average and have witnessed 3.5 events, compared to only 1.2 and 1.6 among Bangladeshi adolescents, respectively (*p* < 0.01 for both). In addition to experiencing and witnessing more traumatic events than Bangladeshi adolescents, the types of traumatic events that Rohingya adolescents experience are more severe than those that Bangladeshi adolescents experience, and they also experience them at much higher rates. Rohingya adolescents are most likely to have experienced being close to death (48.3%), being in a combat situation (41%), and torture (41%). In contrast, Bangladeshi adolescents are most likely to have experienced a serious injury (29%), being close to death (23%) and the unnatural death of a family member or friend (17%) (see Panel A of Supplementary Table S1). Reports of witnessing traumatic events tells a similar story (Panel B of Supplementary Table S1). On the other hand, Bangladeshi adolescents have heard of a higher number of traumatic events (5 compared to 3.7, *p* < 0.01). This naturally follows from the lower rates of experiencing or witnessing events directly, with a range of 21–64% of the Bangladeshi adolescents reporting hearing of traumatic events as their closest proximity (see Panel C of Table S1). In the regression analysis that follows, we focus on experiencing or witnessing traumatic events as explanatory variables of interest.

### 3.2. Regression Results

For the remaining analysis, we focus on the sample of Rohingya adolescents to estimate the relationship between exposure to trauma at two different levels of proximity (experiencing and witnessing) and mental health outcomes.

Panel A of Table 2 presents coefficients from a linear regression of mental health outcomes on standardized indexes of traumatic events experienced and witnessed. Overall, Table 2 shows that experiencing and witnessing traumatic events has a negative impact on adolescents’ mental health. In column 1 of Table 2, a one standard deviation increase in the number of traumatic events experienced (sd = 2.06) is associated with an increase in the PTSD score by 0.085 points, a 5% increase over the mean (*p* < 0.01). Similarly, a one standard deviation increase in the number of traumatic events witnessed (sd = 2.47) is associated with a 0.108 point increase in the PTSD score (*p* < 0.01). Neither experiencing nor witnessing traumatic events are statistically significantly associated with PTSD as binary measure in column 2. Supplementary Table S2 presents regression results looking into the experience and witnessing of traumatic events individually, and shows that experiencing being close to death and experiencing murder of a family or friend have the strongest impact on increasing the burden of mental disorders. Witnessing imprisonment and being close to death are also strongly associated with negative mental health outcomes.

Table 2, columns 3–5, explore the effect of exposure to trauma on the likelihood of exhibiting signs of depression. In column 3, one standard deviation increase in the number of traumatic events experienced is associated with an increase in the PHQ-9 score by 0.792 points, a 14% increase over the mean (*p* < 0.01), and a one standard deviation increase in the number of traumatic events witnessed is associated with an increase in the PHQ-9 score by 0.865 points (*p* < 0.01).

Columns 4 and 5 present evidence that the increase in the PHQ-9 score as a result a one standard deviation increase in the number of traumatic events experienced and witnessed is substantial enough to be associated with increased rates of major depression (3.8 percentage points and 7.8 percentage points, respectively) and increased rates of mild to severe depression symptoms overall (10 percentage points and 7.6 percentage points, respectively). Interestingly, witnessing and experiencing more traumatic events has a similar impact on the likelihood of major depression and of experiencing mild to severe symptoms of depression. Although the coefficient on witnessing traumatic events (0.078) is larger than the coefficient on experiencing traumatic events (0.038) in column 4, these effects are not statistically different from each other (*p* = 0.148).

### 3.3. The Role of Resilience

In Panel B of Table 2, we test resilience as an effect modifier in the relationship between exposure to traumatic events and PTSD and depression. Resilience was found to be a significant modifier only for the relationship between the experience of traumatic events and the likelihood of major depression (Table 2, Panel B, column 4). Having a resilience score on the Child and Youth Resilience measure scale is associated with a reduction in the impact of experiencing traumatic events on the likelihood of major depression by 4.4 percentage points (*p* < 0.01). In fact, this association is strong enough, such that a one standard deviation increase in resilience reduces the total impact of a one standard deviation increase in experiencing traumatic events on the likelihood of major depression to zero.

## 4. Discussion

### 4.1. Principal Findings

Rohingya adolescents living in refugee camps in Bangladesh have experienced and witnessed multiple severe traumatic events, including near death, murder of family and friends, and torture. This exposure is greater both in quantity and severity than traumatic events experienced by a comparable population of Bangladeshi adolescents who are living near the camps. While rates of major depression and PTSD among Rohingya adolescents are not statistically different than those among Bangladeshi adolescents, Rohingya adolescents are 37% more likely to experience any depressive symptoms (55% compared to 40%) than Bangladeshi adolescents, indicating higher occurrence of mental disorders.

Exposure to traumatic events (both witnessing and experiencing) is not statistically significantly related with whether a person qualifies for a clinical diagnosis of PTSD; however, exposure to traumatic events does increase the PTSD score as a continuous outcome. Both continuous and dichotomous measures of depression were found to be significantly associated with exposure to trauma, with experiencing trauma increasing the likelihood of experiencing major depression and any depressive symptoms by 30% and 19%, respectively. The results suggest that witnessing a traumatic event and experiencing a traumatic event have the same impact on the likelihood of depression when compared to experiencing a traumatic event. Finally, we find that adolescents with higher resilience scores have a lower likelihood of developing major depression.

### 4.2. Interpretation of Results

The documentation of exposure to traumatic events and rates of mental disorders among Rohingya adolescents is, in itself, significant. While there has been extensive research on adverse mental health outcomes in refugee populations, very little research has focused on adolescent Rohingya refugees. One example is a study by Riley et al. (2017) that found that the general population of Rohingya refugees has been exposed to a multitude of traumatic events [21]. However, because adolescents are particularly vulnerable, it is important to examine trauma and mental health outcomes specifically for this population. Additionally, there is very little research reporting on adolescent refugee populations living in camps. Many studies focus on resettled refugees in the United States or other Western countries, with very few focused on those still living in camps [30,43].

That exposure to trauma increases the PTSD score but not diagnosis of PTSD indicates that there are refugees in this population who have multiple symptoms of PTSD but who may not have severe enough symptoms to be diagnosed (i.e., high rates of low-level PTSD). Similarly, although there are relatively low rates of major depression among Rohingya adolescents (12.5%), there is a strong relationship between exposure to trauma and experiencing depressive symptoms. These findings suggest that programs focused on targeting adolescents with clinical diagnoses may miss many adolescents living with symptoms of depression and PTSD who could benefit from greater programmatic support. That witnessing a traumatic event and experiencing a traumatic event have the same impact on mental health outcomes may indicate that adolescents are more comfortable reporting witnessing events rather than reporting experiencing them.

Taking all aforementioned findings together suggests a need to improve measurement of mental health and detecting possible exposure to extreme traumatic events among adolescent populations. Such work is being undertaken using children’s drawings to measure mental health outcomes and exposure to violence in refugee settings [44,45].

The role of resilience in the relationship between trauma and depression is a valuable finding. Resilience is defined in the broader literature as a quality of individuals that indicates a capacity to overcome adversity [39]. Findings from this study indicate that resilience is protective against depression, even for high levels of trauma. Because the Child and Youth Resilience Measure (CYRM) explores qualities of the individual and the individual’s environment in their ability to protect against adverse mental health outcomes, this finding creates a significant opportunity for policymakers and public health officials to design interventions that promote environmental changes that potentiate resilience building. These interventions should aim to focus on and address issues within the seven qualitative aspects of resilience that are measured in the CYRM: access to material resources, relationships (with family and peers), identity, power and control, cultural adherence, social justice, and cohesion [39]. Additionally, support and access to education have been found to develop resilience [28]. This finding creates a significant opportunity for policymakers and public health officials to design interventions that promote resilience in order to mitigate the development of depression and other adverse mental health outcomes.

### 4.3. Limitations

As this study draws on a representative sample of Rohingya adolescents living in camps in Cox’s Bazar, Bangladesh, the results of this study may not be generalizable to other adolescent refugee populations because of the unique traumatic experience that the Rohingya people have undergone for generations. Moreover, due to the cross-sectional nature of the data, the results presented here are correlations and should not be interpreted as causal impacts of trauma or resilience on mental health outcomes. Finally, surveys were delivered in Chittagonian for this study. While Chittagonian and the Rohingya and mutually intelligible, there are some differences in words and phrasing that may have obscured the understanding of complex survey questions, such as the Harvard Trauma Questionnaire and measures of our primary outcomes, PTSD and the PHQ-9. To mitigate these errors, enumerators were extensively trained on the delivery of the validated scales, including being provided recordings of the correct delivery.

## 5. Conclusions

This study finds that experiencing and witnessing traumatic events were both found to be significantly associated with increased symptoms of PTSD and depression. These findings are particularly important in the context of Rohingya adolescents, who have endured generations of ethnic persecution. Therefore, the findings of this study may also reflect the effects of intergenerational trauma, as well as recent traumatic events [46]. While this research does not differentiate between the effects of current and intergenerational trauma, it is important to remember that intergenerational trauma may increase vulnerability to exaggerated effects of more recent traumatic events.

The finding that exposure to trauma is associated with increases in continuous PTSD scores and the lack of the same results with PTSD as a dichotomous outcome points to potentially high rates of low levels of PTSD symptoms, with a number of Rohingya refugees at high risk of developing PTSD. This offers an opportunity to develop policies and interventions to focus on preventing these adolescents from developing more severe PTSD symptoms and crossing the threshold into clinical PTSD. Finally, this study’s finding on the protective role of resilience in the relationship between trauma and depression presents an opportunity for the design of interventions that promote resilience building.

## Figures and Tables

**Table 1:** Descriptive statistics, Rohingya refugee and Bangladeshi adolescents aged 15–18.

	(1)	(2)	(3)
	Rohingya	Bangladeshi	*p*-Value
Adolescent age	16.170	16.062	0.221
=if adolescent is female	0.509	0.556	0.326
=1 if enrolled in formal or informal education	0.148	0.622	<0.001
Asset Deciles	6.007	5.753	0.432
PTSD index score (0–3.625)	1.700	1.481	<0.001
=1 if experiencing PTSD (≥2.5)	0.037	0.020	0.253
PHQ-9 index score (0–27)	5.394	4.415	0.004
=1 if moderately depressed (PHQ-9 ≥ 10)	0.125	0.101	0.423
=1 if mild to severe depression (PHQ-9 ≥ 5)	0.547	0.398	0.004
Total events experienced (0–12)	2.757	1.206	<0.001
Total events witnessed (0–12)	3.506	1.592	<0.001
Total events heard (0–12)	3.699	5.014	<0.001
Child and Youth Resilience Measure Total Score	74.296	77.091	<0.001
Observations	361	449	

All means calculated using sampling weights to make them representative of populations in the sampling area. P-values presented in column 3 are from a test of differences of means between the Rohingya and Bangladeshi populations, with standard errors clustered at the respective geographic sampling level (camp-block for Rohingya and sub-mauza for Bangladeshis).

**Table 2:** Relationship between cumulative exposure to trauma and adolescent mental health among Rohingya adolescents.

	(1)	(2)	(3)	(4)	(5)
	PTSD	PTSD (≥2.5)	PHQ-9	PHQ-9 (≥10)	PHQ-9 (≥5)
A. Exposure to traumatic effects					
Experience Traumatic Events Standardized Index	0.085 ***	0.010	0.792 ***	0.038 **	0.104 ***
	(0.025)	(0.012)	(0.217)	(0.018)	(0.037)
Witness Traumatic Events Standardized Index	0.108 ***	0.017	0.865 ***	0.078 ***	0.076 **
	(0.024)	(0.016)	(0.239)	(0.024)	(0.033)
Number of observations	361	361	353	353	353
B. Exploring resilience as a modifier					
Experience Traumatic Events Standardized Index	0.086 ***	0.009	0.802 ***	0.041 **	0.102 ***
	(0.024)	(0.011)	(0.211)	(0.018)	(0.038)
Witness Traumatic Events Standardized Index	0.105 ***	0.015	0.834 ***	0.076 ***	0.068 **
	(0.025)	(0.015)	(0.215)	(0.024)	(0.034)
Experience Traumatic Events Standardized Index × Resilience	−0.018	−0.006	−0.269	−0.044 ***	−0.036
	(0.019)	(0.008)	(0.184)	(0.015)	(0.025)
Witness Traumatic Events Standardized Index × Resilience	−0.011	−0.006	0.028	0.001	0.001
	(0.020)	(0.014)	(0.208)	(0.018)	(0.027)
Resilience Standardized Index	−0.033	−0.010	−0.339 **	−0.012	−0.035
	(0.022)	(0.009)	(0.165)	(0.012)	(0.027)
Number of obervations	344	344	336	336	336

*** *p* < 0.01, ** *p* < 0.05, * *p* < 0.10. Each column is a separate model. All models include controls for adolescent age, gender, school enrollment, asset index, Upazila of camp and sampling considerations. Standard errors clustered at the camp-block level are in parentheses.

## Data Availability

The data preseted in this study are openly available in the UK Data Archive at 10.5255/UKDA-SN-8750-1, study number 8750.

## Supplementary Materials

The following are available online at https://www.mdpi.com/article/10.3390/ijerph18105318/s1, Table S1: Summary Statistics of Traumatic Events by Item and Table S2: Association between individual traumatic events and mental health outcomes among Rohingya Adolescents.
