# Supplementary material for: Displacement, Violence, and Mental Health: Evidence from Rohingya Adolescents in Cox’s Bazar, Bangladesh
Source: Int J Environ Res Public Health. 2021 May 17;18(10):5318. doi: 10.3390/ijerph18105318 (PMC8156348; doi:10.3390/ijerph18105318)
Supplement: Supplementary file 1 [file ijerph-18-05318-s001.zip › ijerph-1147402-supplementary.pdf]

Supplementary Materials for Displacement, Violence, and Mental Health: Evidence from Rohingya Adolescents in Cox's Bazar, Bangladesh

Table S1. Summary Statistics of Traumatic Events by Item.

	(1)	(2)	(3)
	Rohingya	Bangladeshi	p-value
**A. Experience Traumatic Events**			
=1 if Experience imprisonment	0.127	0.103	0.449
=1 if Experience serious injury	0.277	0.290	0.785
=1 if Experience combat situation	0.410	0.151	0.000
=1 if Experience rape or sexual abuse	0.016	0.011	0.613
=1 if Experience isolation	0.275	0.022	0.000
=1 if Experience being close to death	0.483	0.232	0.000
=1 if Experience force separation from family	0.178	0.050	0.000
=1 if Experience murder of family or friend	0.107	0.040	0.015
=1 if Experience murder of stranger(s)	0.107	0.040	0.015
=1 if Experience lost or kidnapped	0.092	0.050	0.073
=1 if Experience torture	0.407	0.043	0.000
=1 if Experience unnatural death of family/friend	0.279	0.174	0.012
**B. Witness Traumatic Events**			
=1 if Witness imprisonment	0.490	0.304	0.000
=1 if Witness serious injury	0.487	0.271	0.000
=1 if Witness combat situation	0.419	0.234	0.000
=1 if Witness rape or sexual abuse	0.253	0.069	0.000
=1 if Witness isolation	0.264	0.078	0.000
=1 if Witness being close to death	0.174	0.141	0.285
=1 if Witness force separation from family	0.119	0.046	0.003
=1 if Witness murder of family or friend	0.355	0.065	0.000
=1 if Witness murder of stranger(s)	0.355	0.065	0.000
=1 if Witness lost or kidnapped	0.246	0.058	0.000
=1 if Witness torture	0.258	0.153	0.006
=1 if Witness unnatural death of family or friend	0.087	0.109	0.410
**C. Heard of Traumatic Events**			
=1 if Heard of imprisonment	0.326	0.468	0.002
=1 if Heard of serious injury	0.187	0.281	0.026
=1 if Heard of combat situation	0.145	0.477	0.000
=1 if Heard of rape or sexual abuse	0.666	0.639	0.575
=1 if Heard of isolation	0.276	0.358	0.058
=1 if Heard of being close to death	0.201	0.287	0.038
=1 if Heard of force separation from family	0.127	0.213	0.014
=1 if Heard of murder of family or friend	0.464	0.583	0.020
=1 if Heard of murder of stranger(s)	0.464	0.583	0.020
=1 if Heard of lost or kidnapped	0.531	0.588	0.233
=1 if Heard of torture	0.233	0.376	0.001
=1 if Heard of unnatural death of family or friend	0.080	0.162	0.009

Notes. All means calculated using sampling weights to make them representative of populations in the sampling area. p-values presented in column 3 are from a test of differences of means between the Rohingya and Bangladeshi populations, with standard errors clustered at the respective geographic sampling level (camp-block for Rohingya and sub-mauza for Bangladeshis).

Table S2. Association between individual traumatic events and mental health outcomes among Rohingya Adolescents.

	(1)	(2)	(3)	(4)	(5)
	PTSD	PTSD (>=2.5)	PHQ-9	PHQ-9 (>=10)	PHQ-9 (>=5)
=1 if Experience imprisonment	-0.120	-0.003	0.127	-0.004	-0.061
	(0.092)	(0.019)	(0.876)	(0.063)	(0.144)
=1 if Experience serious injury	0.034	-0.053	0.164	-0.025	0.131
	(0.078)	(0.033)	(0.709)	(0.059)	(0.106)
=1 if Experience combat situation	-0.147*	-0.031	-0.712	-0.067	-0.082
	(0.077)	(0.037)	(0.610)	(0.059)	(0.085)
=1 if Experience rape or sexual abuse	0.110	0.148	0.586	0.085	0.251
	(0.277)	(0.230)	(1.939)	(0.230)	(0.164)
=1 if Experience isolation	0.129*	0.031	0.471	0.094*	0.046
	(0.068)	(0.034)	(0.558)	(0.053)	(0.082)
=1 if Experience being close to death	0.132*	0.031	1.681***	0.125**	0.134
	(0.069)	(0.032)	(0.580)	(0.051)	(0.093)
=1 if Experience force separation from family	0.122*	-0.050	-0.721	-0.160***	-0.007
	(0.071)	(0.037)	(0.605)	(0.050)	(0.093)
=1 if Experience murder of family or friend	0.135	0.056	1.588**	0.077	0.270**
	(0.091)	(0.056)	(0.661)	(0.069)	(0.116)
=1 if Experience lost or kidnapped	0.111	-0.050	0.025	0.020	0.107
	(0.082)	(0.040)	(0.774)	(0.086)	(0.116)
=1 if Experience torture	0.023	0.040	0.018	0.048	-0.058
	(0.068)	(0.033)	(0.547)	(0.054)	(0.090)
=1 if Experience unnatural death of family or friend	0.039	0.013	1.323**	0.082	0.170**
	(0.069)	(0.041)	(0.579)	(0.064)	(0.078)
=1 if Witness imprisonment	0.084	0.028	1.065**	0.096**	0.004
	(0.054)	(0.027)	(0.488)	(0.041)	(0.076)
=1 if Witness serious injury	-0.000	-0.021	-0.335	-0.057	0.071
	(0.076)	(0.034)	(0.595)	(0.052)	(0.084)
=1 if Witness combat situation	-0.043	-0.018	-0.065	0.006	-0.006
	(0.076)	(0.040)	(0.599)	(0.053)	(0.091)
=1 if Witness rape or sexual abuse	0.062	0.027	0.727	0.043	0.041
	(0.063)	(0.024)	(0.540)	(0.059)	(0.076)
=1 if Witness isolation	-0.028	0.010	-0.624	0.016	-0.082
	(0.060)	(0.022)	(0.523)	(0.052)	(0.079)
=1 if Witness being close to death	0.096	0.078*	2.393***	0.227***	0.199*
	(0.078)	(0.042)	(0.796)	(0.068)	(0.110)
=1 if Witness force separation from family	0.192**	0.060	1.152	0.072	0.143
	(0.096)	(0.054)	(0.828)	(0.089)	(0.105)
=1 if Witness murder of family or friend	0.201***	0.018	0.701	0.045	0.021
	(0.067)	(0.032)	(0.573)	(0.053)	(0.080)
=1 if Witness lost or kidnapped	-0.090	-0.021	-0.344	-0.018	0.035
	(0.072)	(0.029)	(0.656)	(0.060)	(0.094)
=1 if Witness torture	-0.000	-0.025	-0.563	-0.029	-0.088
	(0.067)	(0.020)	(0.627)	(0.065)	(0.091)
=1 if Witness unnatural death of family or friend	-0.029	-0.044	0.278	0.091	0.074
	(0.078)	(0.034)	(0.870)	(0.094)	(0.131)
Number of observations	361	361	353	353	353

Notes. ***p<0.01, **p<0.05, *p<0.10. Each column is a separate model. All models include controls for adolescent age, gender, school enrollment, asset index, upazila of camp and sampling considerations. Standard errors clustered at the camp-block level are in parentheses.
